# Effects of *Kluyveromyces marxianus* supplementation on immune responses, intestinal structure and microbiota in broiler chickens

**DOI:** 10.1371/journal.pone.0180884

**Published:** 2017-07-10

**Authors:** Weiwei Wang, Zhui Li, Zengpeng Lv, Beibei Zhang, Hong Lv, Yuming Guo

**Affiliations:** 1 State Key Laboratory of Animal Nutrition, College of Animal Science and Technology, China Agricultural University, Beijing, P. R. China; 2 Shanghai Engineering Research Center of Industrial Microorganisms, College of Life Sciences, Fudan University, Shanghai, P. R. China; Leibniz-Institut fur Pflanzengenetik und Kulturpflanzenforschung Gatersleben, GERMANY

## Abstract

To investigate the effects of *Kluyveromyces marxianus* on immune responses, intestinal structure and microbiota in broilers, 840 1-d-old broiler chicks were randomly divided into seven groups (eight replicates) and were fed basal diets without or with 0.25, 0.50, 1.0, 1.5, 2.0, and 2.5 g/kg of *K*. *marxianus* (2.0×10^10^ CFU/g). Serum and intestine samples were collected at 21 d of age. The results showed that increasing *K*. *marxianus* addition linearly reduced feed conversion ratio but linearly elevated relative thymus weight, as well as quadratically increased serum lysozyme and IgG levels, with the medium dose (1.0 g/kg) being the most effective. The ratio of villus height to crypt depth of jejunum and ileum, ileal villus height and sucrase activity, as well as the mRNA expression of ileal mucin-2, claudin-1 and sodium glucose cotransporter 1 linearly responded to the increasing *K*. *marxianus* addition. Supplemental *K*. *marxianus* at low (0.5 g/kg), medium (1.5 g/kg) and high (2.5 g/kg) dose all decreased the abundance of phylum *Cyanobacteria*, increased the abundance of phylum *Firmicutes* and genus *Lactobacillus* in ileum. The high dose of *K*. *marxianus* addition also reduced the abundance of order *Rickettsiales* and *Pseudomonadales* along with species *Acinetobacter junii*. Ileal bacterial communities between *K*. *marxianus*-treated and untreated groups formed distinctly different clusters. In summary, *K*. *marxianus* supplementation benefits feed efficiency and immune function, as well as intestinal structure in broilers, which might be attributed to the improved ileal microbial structure. Supplemental *K*. *marxianus* at high dose (2.5 g/kg) was more effective for feed efficiency and intestinal health of broilers, while the innate immunity was optimized at a medium dose (1.0 g/kg).

## Introduction

Yeast probiotic (mainly the *Saccharomyces cerevisiae*) had been widely used in animal production, as it was indicated to improve growth performance, immune responses, and intestinal health in animals [1–4]. This might be due to its prebiotic function and interaction with specific immune cells of host by the cell wall components [1–4]. However, there is paucity of information regarding the effects of other yeast species in animals. *Kluyveromyces marxianus*, a food-grade probiotic, have been approved by Chinese Ministry of Health and European Union. *K*. *marxianus* had gained increasing attention on the commercial production field because of its unique physiological properties such as high growth rate, active metabolic functions, high thermostability and high content of mannan in the cell wall in comparison with *S*. *cerevisiae* [5–7], based on which *K*. *marxianus* addition might be helpful for growth performance and physiological function of animals. Experiments in ruminants have evidenced that *K*. *marxianus* addition had ability to improve feed efficiency and enhance some digestive enzymes activities [8, 9]. In an *in vitro* study, it was suggested that *K*. *marxianus* had immunomodulatory properties as well as a protective role for intestinal barrier function [10–13]. However, it was unknown whether dietary *K*. *marxianus* supplementation could be helpful for immune responses and intestinal structure in chickens.

Gut microbiota has major impact on the bioavailability and bioactivity of dietary components, playing important roles in host nutritional, physiological and protective functions [14, 15]. It is necessary to decipher the content and diversity of intestinal microbial community in order to understand and exploit gut microbiota and the potential influence of its manipulation on performance and health of host. Besides, an understanding and a description of gut microbiota are critical for the development of new feed additives and the appropriate manipulation of diet to improve growth performance and health status in chickens [16]. It was suggested that the improvement of growth performance, immune function and intestinal health in chickens could be linked with the modification of intestinal microbial structure [17–20]. *In vitro*, *K*. *marxianus* was identified as a functional probiotic with associated antibacterial properties and plays a role in modifying microbial composition [10–13]. Nevertheless, it is unknown whether dietary *K*. *marxianus* supplementation can improve intestinal microbial structure in animals, which might subsequently benefit the immune function and intestinal structure of host. Therefore, the present study was conducted to investigate the effects of *K*. *marxianus* supplementation on growth performance, immune responses and intestinal structure in broiler chickens. In addition, we aimed to assess the shifts in intestinal microbial community structure induced by dietary treatment to explain the possible beneficial effects of *K*. *marxianus* addition on broilers.

## Materials and methods

### Birds and experimental design

The experimental animal protocol for this study was approved by the Animal Care and Use Committee of China Agricultural University. A total of 840 one-day-old female Arbor Acre broiler chicks were randomly allocated into 7 treatment groups with 8 replicates of each. Each replicate pen involving 15 birds. Initial body weights were similar across all the replicates. Birds received basal diets in mash form without or with 0.25, 0.50, 1.0, 1.5, 2.0, 2.5 g/kg *K*. *marxianus* (*K*. *marxianus* FIM-1, 2.0×10^10^ CFU/g) throughout the trial period. The product was provided by Shanghai Engineering Research Center of Industrial Microorganisms (China), and has been deposited in China General Microbiological Culture Collection Center (CGMCC) with a reference number of 10621. The composition of basal diets are shown in Table 1. All birds were raised in wired three-level battery cages (100 cm long × 80 cm wide × 40 cm high/cage). Feed and fresh water were available *ad libitum*. The lighting schedule was 20 h light and 4 h dark throughout the experiment. The room temperature was controlled with heaters and gradually reduced from 35°C on d 1 to 24°C on d 21 and then kept roughly constant. Birds were vaccinated using combined Newcastle disease virus (NDV) and infectious bronchitis virus on d 7 through intranasal and intraocular administration, and on d 21 via oral administration.

**Table 1:** Composition and nutrient levels of diets (g/kg).

Ingredients	Stage	
	1−21 d	22−35 d
Maize	559.7	613.7
Soybean meal (43%, crude protein)	376.3	318.8
Soybean oil	23.7	31.3
Limestone	12.7	12.1
Sodium chloride	3.5	3.5
Dicalcium phosphate	17.5	14.9
Choline chloride (50%)	2.0	2.0
DL-Methionine (98%)	2.0	1.2
L-Lysine·HCl (99%)	0.2	0.1
Antioxidant	0.2	0.2
Multivitamin^1^	0.2	0.2
Multimineral^2^	2.0	2.0
Yeast^3^	+**/-**	+/-
Nutrient levels		
Metabolic Energy (MJ/kg)	12.14	12.56
Crude Protein	210.0	190.0
Available Phosphorus	4.5	4.0
Calcium	10.0	9.0
Lysine	11.5	10.0
Methionine	5.0	4.0

^1^ Supplied per kg of diet: retinyl acetate, 24 mg; cholecalciferol, 6 mg; menadione, 2.65 mg; thiamin, 2 mg; riboflavin, 6 mg; cyanocobalamin, 0.025 mg; α-tocopherol acetate, 20 mg; biotin, 0.0325 mg; folic acid, 1.25 mg; pantothenic acid, 12 mg; niacin, 50 mg.

^2^ Supplied per kg of diet: Cu, 8 mg; Zn, 75 mg; Fe, 80 mg; Mn, 100 mg; Se, 0.15 mg; I, 0.35 mg.

^3^ Yeast (*Kluyveromyces marxianus*, the measured value of clump count was 1.5×10^10^ CFU/g) was substituted for the same amount of maize.

### Sample collection and procedure

Birds were randomly selected from each replicate pen (8 birds per group) on d 21 for samples collection. Individual blood samples were taken aseptically from the wing vein. Serum samples were separated by centrifugation of blood at 3000 rpm for 10 min at 4°C and stored at −30°C until analysis. After blood collection, these birds were slaughtered through cervical dislocation. Spleen, thymus and bursa from each bird were excised and weighed to determine the relative weight of immune organs, which were expressed as the ratio of organ weight (g) to body weight (kg). Mid-segments of jejunum and ileum were harvested and cut into two sections: one of which was fixed in 4% paraformaldehyde solution for morphology measurement, and the other was put into liquid nitrogen and kept at −80°C for quantification of gene expression. Meanwhile, digesta and mucosa from the remainder intestine were collected and quick-freezed using liquid nitrogen, followed by storage at −80°C until further analysis.

### Performance measurement

Body weight and feed intake were recorded for each replicate at 21 and 35 d of age. Average body weight (ABW) at 21 and 35 d of age, along with average daily gain (ADG), average daily feed intake (ADFI), and feed conversion ratio (FCR) during the grower period (1−21 d) and the overall period (1−35 d) were calculated.

### Biochemical assay of serum and intestinal mucosa

Activity of serum lysozyme was determined colorimetrically under the instruction of a commercial kit (Jiancheng Biotechnology Institute, Nanjing, China). Concentrations of serum IgG, IgM, and IgA as well as intestinal secretory IgA (sIgA) were assayed by double-antibody sandwich ELISA using commercial kits (Bethyl Laboratories Inc., Montgomery, TX, USA) according to the manufacturer′s instructions. Intestinal alkaline phosphatase (ALP) activity was measured by colorimetric assay using a corresponding diagnostic kit (Jiancheng Biotechnology Institute, Nanjing, China) under the manufacturer′s protocols. Activity of intestinal disaccharidase was determined as described previously [21]. The results of above_–_mentioned intestinal indicies were normalised by total protein content, which was determined using BCA protein quantitation kits (CWBiotech Co. Ltd, Beijing, China).

### Intestinal morphological analysis

The 2-μm cross_–_sections of jejunal and ileal tissues were obtained after staining with hematoxylin-eosin using standard paraffin-embedding procedures. For each section, ten representative intact villi were selected for morphology examination using Leica DMI6000B light microscope equipped with an image-processing software (Leica application suite V4.2). Villus height (VH) was determined from the tip of villus to the junction of villus and crypt, crypt depth was defined as the depth of emboly between adjacent villi, villus height to crypt depth ratio (VCR) was calculated. The mean value of these ten values represents the final value of each sample.

### Quantification of mRNA expression of intestinal genes

Total RNA were extracted from the ileum using Trizol Reagent (Invitrogen biotechnology Inc., Carlsbad, USA). Extracted RNA was dissolved in RNase-free water and quantified using Nanodrop spectrophotometer (ND-2000 UV-Vis; Thermo Scientific Inc., USA). RNA Purity was verified by determining the absorbance ratio at 260:280 nm. RNA integrity was evaluated on the basis of the spectral curve [22]. The cDNA samples were obtained by reverse transcription of total RNA using PrimeScript^TM^ RT reagent kit with gDNA Eraser (Takara Biotechnology Inc., Osaka, Japan) and were kept at –20°C until analyzed. Real-time PCR for measuring intestinal genes expression was carried out using SYBR^®^ Premix Ex Taq^TM^ (Tli RNaseH Plus) (Takara Biotechnology Inc., Osaka, Japan) in ABI 7500 Real Time PCR Systems (Applied Biosystems, Foster City, California, USA). The expression of β-actin was used as an internal control to normalise the amount of initial RNA for each sample. The reaction volume of 20 μl mixture contained 10 μl SYBR^®^ Premix Ex Taq (Tli RNaseH Plus), 0.4 μl ROX Reference Dye, 0.4 μl of each forward and reverse primer, 6.8 μl dilution and 2 μl cDNA template. Primer sequences for the target and reference genes are shown in Table 2. The protocol for all these genes were 95°C for 30 s, followed by 40 cycles of 95°C for 5 s and 60°C for 34 s. All measurements were carried out in duplicate. PCR efficiency for each gene was figured out according to the slope of cDNA relative standard curve that was generated using pooled samples. The efficiency values between the reference and target genes were consistent. The abundance of β-actin mRNA was not influenced by dietary treatment. Specificity of PCR products was evaluated by the analysis of melting curve. The results of relative mRNA expression of intestinal genes were calculated using the 2^-ΔΔCt^ method [23].

**Table 2:** Primers used in the real-time PCR.

Genes^1^	Primer sequence^2^ (5′-3′)	Accession no.
*β-Actin*	F: GAGAAATTGTGCGTGACATCA	L08165
	R: CCTGAACCTCTCATTGCCA	
*Mucin-2*	F: TTCATGATGCCTGCTCTTGTG	XM_421035
	R: CCTGAGCCTTGGTACATTCTTGT	
*Claudin-1*	F: CATACTCCTGGGTCTGGTTGGT	AY750897.1
	R: GACAGCCATCCGCATCTTCT	
*SGLT1*	F: GATGTGCGGATACCTGAAGC	AJ236903
	R: AGGGATGCCAACATGACTGA	
*PepT1*	F: TACGCATACTGTCACCATCA	AY029615
	R: TCCTGAGAACGGACTGTAAT	
*L-FABP*	F: GAAGGGTAAGGACATCAA	NM_204192
	R: TCGGTCACGGATTTCAGC	

^1^*SGLT1*, sodium glucose cotransporter 1; *PepT1*, H+-dependent peptide transporter 1

*L-FABP*, liver fatty acid-binding protein.

^2^ F, forward; R, reverse.

### Pyrosequencing of ileal microbiota

DNA samples were extracted from ileal digesta using QIAamp DNA Stool Mini Kits (Qiagen Inc., Hilden, Germany) according to the manufacturer′s instructions. The concentration and quality of DNA samples were checked with gel electrophoresis and a Nanodrop 2000 spectrophotometer (Thermo Scientific Inc., Waltham, USA). Bacterial 16S rDNA sequences spanning the variable regions V3–V4 were amplified using primer 515 F (5′-GTG CCA GCM GCC GCG GTA A-3′) and 806 R (5′-GGA CTA CHV GGG TWT CTA AT-3′). All PCR were carried out in 30-μl reaction volumes containing 15 μl of Phusion High-Fidelity PCR Master Mix (New England Biolabs, Ipswich, MA, USA), 0.2 μM forward and reverse primers, and 10 ng template DNA. Amplification by PCR consisted of the following: initial denaturation at 98°C for 1 min, followed by 30 cycles at 98°C for 10 s, 50°C for 30 s, and 72°C for 30 s, and a final extension step at 72°C for 5 min. PCR products were detected by 2% agarose gel electrophoresis and purified with QIAquick Gel Extraction Kit (Qiagen Inc., Hilden, Germany). A library was constructed using TruSeq^®^ DNA PCR-Free Sample Preparation Kit (Illumina, San Diego, USA) and detected by Qubit and q-PCR quantification. Pyrosequencing for 16S rDNA was carried out on the Illumina HiSeq2500 PE250 platform (Illumina, San Diego, USA). All of the procedures were conducted by Novogene Bioinformatics Technology Co. Ltd. (Beijing, China). Sample reads were assembled using Mothur software. Clustering of filtered sequences into operational taxonomic units (OTUs) was achieved using Uparse at 97% sequence identity. Taxonomic classification at different taxonomic levels of these OTU sequences was performed by comparing sequences to the GreenGene database. Qiime software and Python scripts were used for the analysis of microbial diversity. The Unifrac approach was used to estimate pairwise distances between samples and to establish beta diversity, which was visualized by principal component analysis (PCA) and clustering analysis.

### Statistical analysis

Data were presented as mean ± standard error of the mean (SEM). Pens were used as the experimental unit for growth performance parameters, whereas an individual bird served as the experimental unit for other parameters. The polynomial regression analysis (SPSS 18.0) was used to test the linear and quadratic nature of the response to the additive dosage of *K*. *marxianus*. Significance was defined as *P* < 0.05 and 0.05 < *P* < 0.10 was considered to be a tendency towards significance.

## Results

### Growth performance

Dietary *K*. *marxianus* supplementation had no effect (*P* > 0.05) on ABW of birds at 21 or 35 d of age (Table 3), as well as ADG and ADFI during grower period and the overall period. However, with the increase of *K*. *marxianus* addition, FCR during grower period and the overall period exhibited positive linear response (*P* < 0.05), with greater levels (2.0 and 2.5 g/kg) being more effective.

**Table 3:** Effects of *Kluyveromyces marxianus* on growth performance^1^ of broilers.

Dose (g/kg)	21 d	1−21 d			35 d	1−35 d		
	ABW	ADG	ADFI	FCR	ABW	ADG	ADFI	FCR
0	673	29.95	42.09	1.406	1683	46.89	77.14	1.645
0.25	693	30.85	43.25	1.403	1723	47.33	77.83	1.645
0.50	697	31.11	43.32	1.393	1734	47.92	78.17	1.632
1.0	678	30.18	41.43	1.373	1695	47.06	76.05	1.617
1.5	715	31.96	43.96	1.377	1760	48.91	78.90	1.614
2.0	688	30.67	41.80	1.364	1724	47.40	76.25	1.609
2.5	699	31.17	42.60	1.367	1703	47.50	76.25	1.606
SEM	4.5	0.211	0.255	0.0046	7.7	0.232	0.425	0.0065
*P*-value								
Linear	0.196	0.204	0.753	0.001	0.547	0.445	0.346	0.024
Quadratic	0.299	0.318	0.890	0.002	0.182	0.330	0.518	0.064

*n* = 8 replicates per group.

^1^ABW, average body weight (g); ADG, average daily gain (g); ADFI, average daily feed intake (g); FCR, feed conversion ratio.

### Relative weight of immune organs and serum parameters

There were no changes (*P* > 0.05) in the relative weight of spleen and bursa, as well as serum IgA and IgM levels in response to *K*. *marxianus* addition (Table 4). However, there was a linear increase (*P* < 0.05) in the relative thymus weight responded to the increasing *K*. *marxianus* addition. Responses to increasing *K*. *marxianus* addition on serum lysozyme and IgG levels were quadratic (*P* < 0.05), with the medium dose (1.0 g/kg) being the most effective.

**Table 4:** Effects of *Kluyveromyces marxianus* on the relative weight^1^ of immune organs and serum parameters of broilers (21 d of age).

Dose (g/kg)	Spleen	Bursa	Thymus	IgG mg/mL	IgA mg/mL	IgM mg/mL	Lysozyme U/mL
0	0.92	2.12	2.68	1.65	0.38	0.30	171.24
0.25	0.83	2.15	2.68	1.85	0.44	0.33	194.57
0.50	0.80	2.04	2.76	2.53	0.33	0.36	185.62
1.0	0.86	1.99	2.90	3.14	0.40	0.34	204.97
1.5	0.82	1.94	2.88	2.46	0.41	0.23	204.57
2.0	0.79	1.96	2.98	2.31	0.39	0.24	202.51
2.5	0.87	2.22	3.42	2.89	0.45	0.27	185.90
SEM	0.022	0.059	0.069	0.144	0.024	0.019	3.632
*P*-value							
Linear	0.602	0.923	0.001	0.044	0.507	0.103	0.228
Quadratic	0.483	0.344	0.003	0.040	0.705	0.334	0.016

*n* = 8 replicates per group.

^1^ Relative weight of immune organs was expressed as the ratio of organ weight (g) to body weight (kg).

### Intestinal biochemical parameters and morphological structure

*K*. *marxianus* supplementation had no influence (*P* > 0.05) on sIgA content, as well as ALP and maltase activities in the jejunum and ileum (Table 5), but exerted positive linear effect (*P* < 0.05) on ileal sucrase activity. No difference (*P* > 0.05) was noted in jejunal or ileal crypt depth of broilers after *K*. *marxianus* supplementation (Table 6). However, with the increase of *K*. *marxianus* addition, the VCR of jejunum and ileum coupled with ileal VH linearly increased (*P* < 0.05).

**Table 5:** Effects of *Kluyveromyces marxianus* on intestinal biochemical indicies^1^ of broilers (21 d of age).

Dose (g/kg)	Jejunum				Ileum			
	sIgA	AKP	Suc	Mal	sIgA	AKP	Suc	Mal
0	3.02	0.87	136.78	295.59	5.36	0.68	166.97	242.86
0.25	3.35	1.01	154.72	294.18	5.00	0.71	174.62	232.40
0.50	2.89	0.86	148.89	334.73	4.05	0.61	195.19	216.91
1.0	2.60	1.18	143.59	299.85	5.64	0.61	191.43	237.95
1.5	3.37	0.96	136.87	290.13	5.88	0.63	206.98	267.28
2.0	3.82	1.13	129.41	292.23	6.11	0.62	198.64	251.95
2.5	3.26	0.90	154.50	279.11	5.57	0.66	208.67	219.54
SEM	0.179	0.040	4.517	5.890	0.216	0.014	5.345	5.709
*P-*value								
Linear	0.332	0.550	0.848	0.153	0.087	0.347	0.018	0.765
Quadratic	0.612	0.195	0.796	0.237	0.223	0.165	0.040	0.407

*n* = 8 replicates per group.

^1^ sIgA, secretory Ig A (mg/g protein); AKP, alkaline phosphatase (U /mg protein); Suc, sucrase (U/mg protein); Mal, maltase (U/mg protein).

**Table 6:** Effects of *Kluyveromyces marxianus* on intestinal morphology^1^ of broilers (21 d of age).

Dose (g/kg)	Jejunum			Ileum		
	VH	CD	VCR	VH	CD	VCR
0	938.16	140.57	7.03	574.59	97.37	6.30
0.25	912.11	145.67	6.72	613.58	106.01	6.08
0.50	895.90	131.04	7.20	595.66	99.03	6.23
1.0	975.95	142.51	7.39	621.17	106.91	6.24
1.5	1014.85	147.69	7.33	666.40	104.74	6.89
2.0	1022.13	140.05	7.72	643.50	95.25	7.14
2.5	981.88	133.02	7.71	659.47	109.27	6.55
SEM	23.258	3.497	0.149	12.763	2.269	0.131
*P*-value						
Linear	0.137	0.745	0.043	0.033	0.604	0.042
Quadratic	0.289	0.768	0.131	0.085	0.874	0.111

*n* = 8 replicates per group.

^1^ VH, villus height (μm); CD, crypt depth (μm); VCR, the ratio of VH:CD.

### Relative mRNA expression of intestinal genes

*K*. *marxianus* supplementation did not affect (*P* > 0.05) the relative expression of ileal liver fatty acid-binding protein *(L-FABP)* and H^+^-dependent peptide transporter 1 (*PepT1*) (Table 7), but linearly increased (*P* < 0.05) the expression of mucin-2, claudin-1, and sodium glucose cotransporter-1 (*SGLT-1*) in the ileum.

**Table 7:** Effects of *Kluyveromyces marxianus* on the relative mRNA expression of ileal genes^1^ of broilers (21 d of age).

Dose (g/kg)	Mucin-2	Claudin-1	L-FABP	PepT-1	SGLT-1
0	1.03	1.06	1.11	1.17	0.95
0.25	0.96	0.91	1.19	0.96	1.05
0.50	0.93	1.14	0.91	1.30	0.93
1.0	1.21	1.09	1.12	1.09	1.07
1.5	1.20	1.07	0.97	0.97	1.04
2.0	1.11	1.24	0.96	0.98	1.15
2.5	1.26	1.49	1.46	1.19	1.21
SEM	0.032	0.076	0.091	0.105	0.038
*P*-value					
Linear	0.003	0.006	0.652	0.886	0.038
Quadratic	0.011	0.004	0.427	0.928	0.105

*n* = 8 replicates per group.

^1^ L-FABP, liver fatty acid-binding protein; PepT-1, H+-dependent peptide transporter 1; SGLT-1, sodium glucose cotransporter 1.

### Microbial community structure of ileum

The 16S rDNA sequencing was used to analyze the ileal microbial community structure of birds in the control group and *K*. *marxianus*-treated groups at three different doses (0.5, 1.5, 2.5 g/kg). Rarefaction curves for the observed OTUs approach a plateau, indicating that sequencing depth was sufficient for the coverage of all OTUs present in ileal samples (S1 Fig). Good′s coverage indice, used to estimate the percentage of total bacterial OTUs represented in a sample, were over 99%, suggesting the 16S rDNA results from each library represented the majority of bacteria in broiler ileum. Notably, *K*. *marxianus* supplementation did not influence alpha diversity (Shannon, Simpson, ACE, and Chao1 indexes) of ileal microbiota (S1 Table).

**Fig 1:**
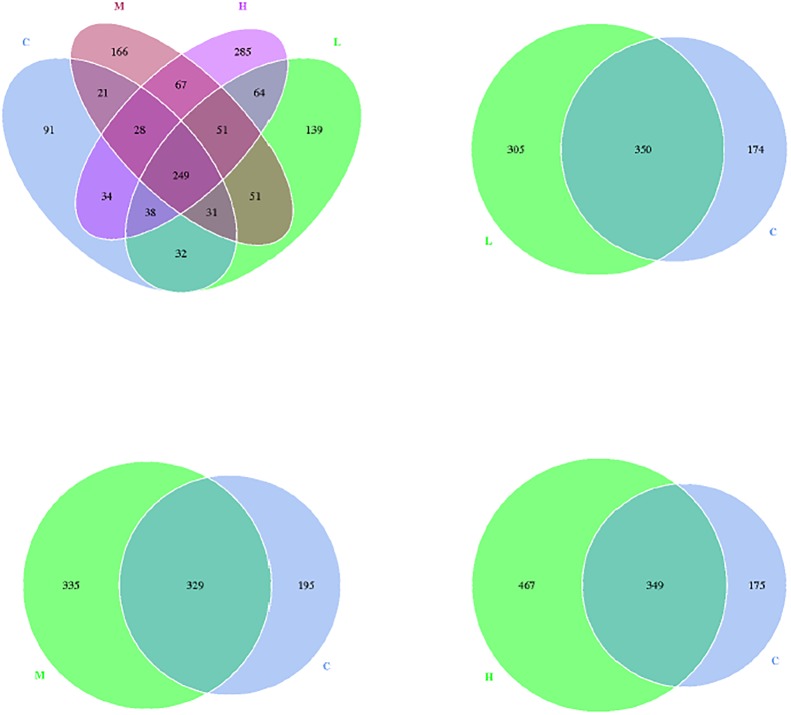
Venn graph for ileal microbiota of broilers at 21 d of age (*n* 8). Each circle represents a group of samples. The numbers of common bacterial operational taxonomic units (OTUs) are displayed in the overlapping section between different circles, while the numbers in the non-overlapping section between different circles represent the number of their respectively unique OTUs. C, control group; L, low-dose (0.5 g/kg) group with *K*. *marxianus*; M, medium-dose (1.5 g/kg) group with *K*. *marxianus*; H, high-dose (2.5 g/kg) group with *K*. *marxianus*.

**Fig 2:**
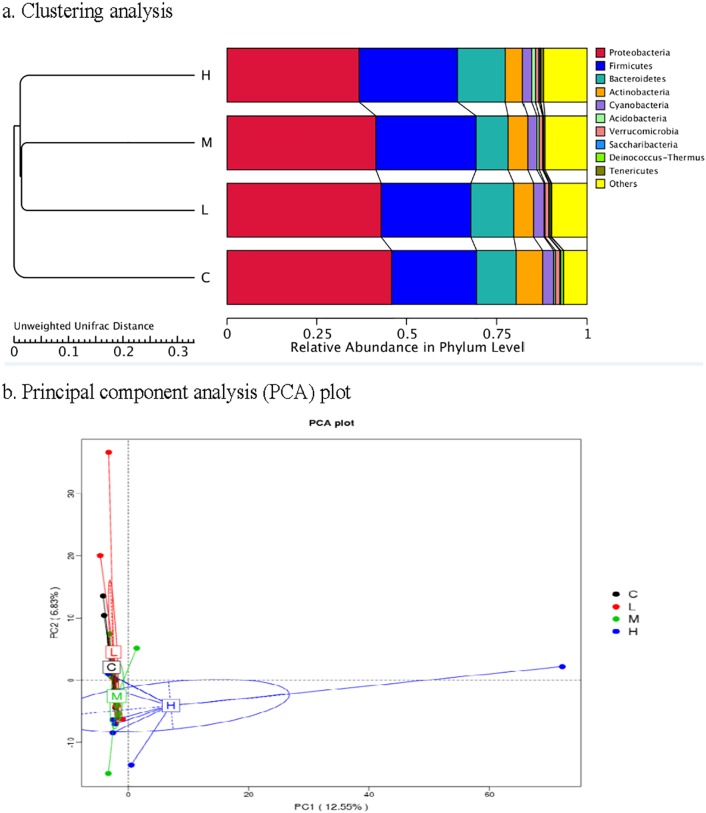
Similarity analysis of ileal microbiota between the control and *Kluyveromyces marxianus*-treated birds (*n* 8). a. Clustering analysis: the left is UPGMA (unweighted pair-group method with arithmetic mean) clustering tree structure, while the relative abundance of main taxa at phylum taxonomic level is displayed on the right. b. PCA: abscissa represents the first principal component, ordinate represents the second principal component, and the percentage represents the contribution of the principal component to the sample difference. C, control group; L, low-dose (0.5 g/kg) group with *K*. *marxianus*; M, medium-dose (1.5 g/kg) group with *K*. *marxianus*; H, high-dose (2.5 g/kg) group with *K*. *marxianus*.

The beta diversity analysis was used to estimate the similarity among different groups. The prevailing occurrence of a common core of shared taxa is exhibited in detail by the overlapping Venn diagrams in Fig 1. The low-dose treatment and the control groups shared 350 bacterial OTUs, while the medium-dose treatment and control groups shared 329 bacterial OTUs, and 349 OTUs were shared by the high-dose treatment and control groups. There were 305, 335 and 467 unique OTUs of ileal microbiota in the low-, medium- and high-dose groups, respectively. The clustering analysis revealed short Unifrac distances among these three *K*. *marxianus* groups and long branches separating the samples from the control and *K*. *marxianus*-treated groups (Fig 2A). The dissimilarity in the ileal microbial community was greatest between the control and high-dose groups. Similarly, principal component analysis (PCA) defined groups where the samples from the control and high-dose groups occupied distinct positions (Fig 2B).

**Fig 3:**
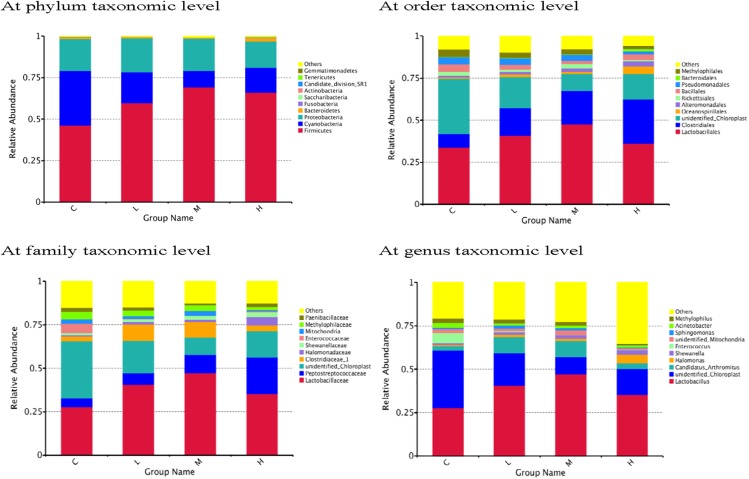
Effects of *Kluyveromyces marxianus* on the relative abundance of main taxa (top ten) in the ileum at different taxonomic levels of broilers (*n* 8). C, control group; L, low-dose (0.5 g/kg) group with *K*. *marxianus*; M, medium-dose (1.5 g/kg) group with *K*. *marxianus*; H, high-dose (2.5 g/kg) group with *K*. *marxianus*.

**Fig 4:**
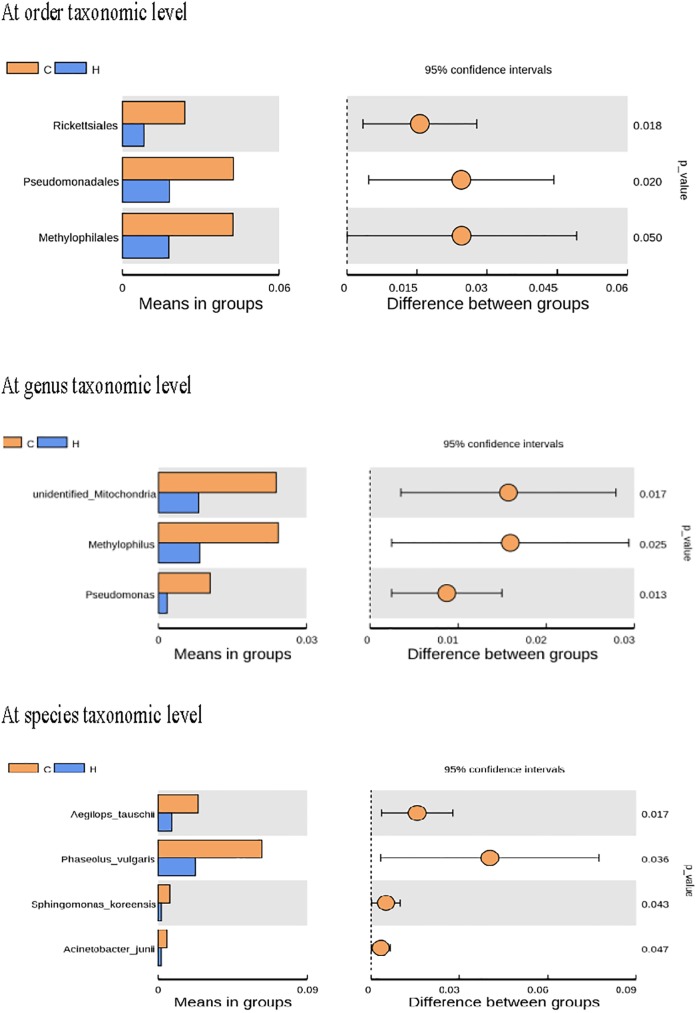
Differentiated species between the control and high-dose group with *Kluyveromyces marxianus* (*n* 8). Mean proportions, 95% confidence intervals and *P* values are represented for each taxon for the two groups of birds. T-tests were used when comparing the relative abundance of individual taxa between different groups. C, control group; H, high-dose (2.5 g/kg) group with *K*. *marxianus*.

The dominant phyla in the ileum across all the groups were the *Firmicutes*, *Cyanobacteria* and *Proteobacteria*, together accounting for more than 90% of the total sequences (Fig 3). Birds supplemented with *K*. *marxianus* at these three levels had higher relative abundance of *Firmicutes* and lower relative abundance of *Cyanobacteria* in the ileal microbiota. However, the abundance of *Proteobateria* was flat among all the groups. Order level microbiota analysis revealed that the ileal microbiota was dominated by *Lactobacillales* and *Clostridiales*, the latter of which was increased in *K*. *marxianus*-treated groups as compared to the control group. Family level microbiota analysis revealed that the ileal microbiota was dominated by *Lactobacteriaceae* and *Peptostreptococcaceae*, which were increased after *K*. *marxianus* treatment. Besides, the *Halomonadaceae* abundance was increased especially in the high-dose group relative to control group. At genus level, an increase in the abundance of *Lactobacillus* was also detected in response to *K*. *marxianus* addition. Further analysis revealed that high-dose group had lower abundance of *Rickettsiales*, *Pseudomonadales* (*Pseudomonas* genus in particular), *Methylophilale* (*Methylophilus* genus in particular), *Acinetobacter junii*, *Sphingomonas koreensis*, *Phaseolus vulgaris* and *Aegilops tauschii* in the ileum than those in the control group (Fig 4).

## Discussion

Live yeast (mainly the *S*. *cerevisiae*) supplementation has been reported to improve the growth performance in animals [1–4]. However, little information is available regarding the effects of *K*. *marxianus* addition in chickens. Similar to the study in ruminants [8], we found that increasing *K*. *marxianus* addition linearly reduced FCR during grower and the overall period of birds, revealing a role for *K*. *marxianus* addition, especially at high dose (2.0 and 2.5 g/kg), in improving the feed efficiency in broiler chickens.

Determination the relative weight of immune organs including bursa of Fabricius, thymus and spleen is a common method for evaluation of immune status in chickens. Increased weight of immune organs in developing healthy animals indicated an increased immunological organ development, which could be beneficial for the immune function of chickens [24]. A previous study indicated that live yeast (*S*. *boulardii*) addition increased the relative weight of thymus and bursa in broilers [25]. Similarly, this study showed that relative weight of thymus was linearly increased by increasing *K*. *marxianus* addition, indicating that *K*. *marxianus* may have led to improved thymus development and subsequently benefit the immunity of broilers. Immunoglobulins bind to antigenic epitopes are critical for humoral immunity. Lysozyme that produced by the phagocytes can disintegrate the polysaccharide walls of Gram-positive and negative bacteria, which is important in innate immune response [26, 27]. Beneficial effects of live yeast (*S*. *cerevisiae*) addition on serum immunoglobulins and lysozyme were observed in animals due to its interaction with specific immune cells of host [25, 26, 28, 29]. In this study, serum IgG level and lysozyme activity quadratically responded to increasing *K*. *marxianus* addition and peaked at the dose of 1.0 g/kg, indicating a stimulation of immune system by *K*. *marxianus* addition especially at a medium dose, thereby providing protection against pathogen invasion in broiler chickens.

Mucosal immunity against infection is mainly mediated by the action of sIgA, which can block the connection between pathogens and epithelium, thereby protecting the intestinal epithelium from pathogenic microorganisms and enteric toxins [30]. Recent studies have shown that live yeast (*S*. *cerevisiae* or *S*. *boulardii*) addition increased intestinal sIgA level in broiler chickens [25, 31]. However, in this study, *K*. *marxianus* addition did not obviously influence the jejunal and ileal sIgA content, which implied different physiological roles of *K*. *marxianus* from other yeast species. Intestinal brush border enzymes that can be classified structurally as intrinsic and extrinsic to the membrane are important for nutrients absorption and gut barrier function [32]. ALP is an intrinsic enzyme in brush border, and serves as an indicator of functional and mature enterocytes in the gut [33]. Disaccharidase is an extrinsic enzyme, which participates in digestion and absorption of nutrients and can also function as a marker for evaluating intestinal mucosal integrity [21]. Few studies were available regarding the effect of live yeast on brush border enzymes in animals. In the present study, though *K*. *marxianus* addition did not influence intestinal ALP activity, ileal sucrase activity was linearly increased by increasing addition of *K*. *marxianus*. This implied that *K*. *marxianus* addition could be, to a degree, helpful for intestinal absorption and integrity of broilers. In support of this view, we also observed an improved intestinal morphology in broilers supplemented with *K*. *marxianus*, as evidenced by the linear increase in VCR of jejunum and ileum and ileal VH in response to *K*. *marxianus* addition. This was similar to some previous studies, in which live yeast (*S*. *cerevisiae* and *S*. *boulardii*) addition improved intestinal morphological structure in both pigs and broilers [1, 2, 25]. As an interface between the host and the environment, intestinal villi perform special functions to maintain intestinal absorption and integrity [21, 34]. Accordingly, the improved intestinal morphology indicated an enhancement of intestinal barrier and absorption capacity of broilers fed with *K*. *marxianus*, which might result in better feed efficiency.

The mucus layer is the first line of defense encountered by intestinal bacteria. Mucin-2 is the primary mucin gene in the mucus layer in the small intestine [35]. Studies pertaining to the effect of live yeast on intestinal mucin profile are sparse. In the present study, increasing *K*. *marxianus* addition linearly increased ileal mucin-2 gene expression, suggesting the idea of an enhanced mucosal barrier function after *K*. *marxianus* addition especially at high dose (2.5 g/kg). This might contribute to the improvement of intestinal morphological structure because of the less bacterial stimulation, and could be beneficial for intestinal immunity since mucin also helps to localize sIgA to the gut epithelium [36]. Another essential component of intestinal barriers are tight junctions (TJs), which create a protective barrier that aids in absorption while preventing translocation of harmful molecules [37]. TJs are comprised of several unique proteins, such as claudin-1 that plays a key role in the action of TJs [38]. This study revealed a linear increase in claudin-1 expression in response to increasing *K*. *marxianus* addition, which was similar to the report of Rajput *et al*. [25], who found that live yeast (*S*. *boulardii*) addition increased TJs proteins expression of broilers. Increased TJs protein expression could result in an enhancement of intestinal barrier function [39], which subsequently reduce the diffusion of macromolecules such as bacterial toxin and pathogens from intestinal lumen into blood circulation, thereby promoting the intestinal and systemic health of host. Nutrient transporters that loaded in the brush border membrane of intestine are greatly important for nutrients absorption. *L-FABP* that expressed in liver and intestine is responsible for the metabolism and intracellular transportation of lipids [40]. *PepT-1* participates in the transportation of peptides into epithelial cells [41]. *SGLT-1* mediates Na^+^-dependent glucose absorption across the luminal membrane of animal enterocytes, which is the major route for the utilisation of dietary sugars [42]. It was reported that probiotic addition increased some types of intestinal nutrient transporters in broilers [43]. An *in vitro* study revealed that *S*. *boulardii* treatment did not markedly enhance H^+^-dependent peptide absorption but promoted Na^+^-dependent glucose absorption of porcine intestinal epithelium [44]. Herein, we found that increasing *K*. *marxianus* addition linearly increased ileal *SGLT-1* expression, which suggested an enhancement of glucose (energy) absorption of birds supplemented with *K*. *marxianus* especially at high dose and may subsequently benefit the growth performance. Therefore, the enhanced intestinal absorption, as characterized by the improved intestinal morphology coupled with the elevated *SGLT-1* expression, could also be related to the improved feed efficiency of broilers after *K*. *marxianus* addition.

Gut microbiota contribute to maintenance of the normal physiological function of intestine, providing a series of beneficial effects on their hosts, such as nutrient digestion, immune function, and protection from invasive pathogens [14–17]. In the present study, we found a high similarity in the ileal microbial community among the broilers treated with different doses of *K*. *marxianus* through PCA and clustering analysis. In contrast, there was a distinct difference between these *K*. *marxianus*-treated and control groups, suggesting that supplemental *K*. *marxianus*, especially at high dose (2.5 g/kg) shifted intestinal microbial composition of broilers. This was supported by Venn diagram analysis, which revealed the largest number of unique bacterial OTUs in the ileal microbiota of birds fed with high-dose of *K*. *marxianus*. The taxonomical composition analysis revealed an enrichment of the phylum *Firmicutes* and a reduction of the phylum *Cyanobacteria* in the ileum after *K*. *marxianus* addition. *Firmicutes* play important roles in polysaccharide decomposition and consequently contribute to the utilisation of dietary energy and intestinal health [45–47]. Also, the reduced feed conversion ratio of broilers was accompanied by the increased abundance of *Firmicutes* [17, 48], which implied a positive correlation between *Firmicutes* abundance and feed efficiency. *Cyanobacteria* can produce cyanotoxins that induce oxidative stress and apoptosis of somatic cells and pose a danger to human health after accumulation through the food chain and drinking water [49, 50]. Therefore, the increase in *Firmicutes* and the reduction of *Cyanobacteria* in the ileum could be related to the improved feed efficiency and intestinal structure of broilers after *K*. *marxianus* addition. At the order level, increases in the abundance of *Clostridiales* and *Lactobacillales* (*Lactobacillus* genus in particular) were observed after *K*. *marxianus* addition. *Clostridiales* was indicated to be associated with the degradation of cellulose in gut [51]. This might be also beneficial for dietary energy utilisation and consequently contribute to the improved feed efficiency of broilers. *Lactobacillus*, as a typical probiotic bacterium, have been reported to modulate immune responses in animals [52, 53]. It could thus be speculated that the increased abundance of *Lactobacillus* as a result of *K*. *marxianus* addition may also be involved with the improved innate immunity of broilers. Among the bacterial species with low abundance, we also found that *K*. *marxianus* addition at high dose decreased the abundance of *Rickettsiales*, *Pseudomonadales* and *Acinetobacter junii*, which are defined as pernicious gut microorganisms that induce a variety of disorders of host [54–57]. Thus, it could be assumed that there was a connection between the reduced harmful germs in gut and the improved intestinal health of birds after *K*. *marxianus* addition at high dose.

## Conclusions

Dietary *K*. *marxianus* supplementation was beneficial for feed efficiency, host immunity, and intestinal structure of broiler chickens. The host immune-related indices (serum IgG and lysozyme levels) of broilers optimized at medium dose (1.0 g/kg) of *K*. *marxianus* addition. However, the responses to *K*. *marxianus* addition on feed conversion ratio and intestinal structure were linearly. Supplemental *K*. *marxianus* especially at high dose (2.5 g/kg) improved intestinal microbial structure, which may contribute to the improved feed efficiency and intestinal structure of broiler chickens.

## Supporting information

S1 Fig Rarefaction curves and Good’s coverage indices of ileal microbial sequencing (*n* 8). (a) Rarefaction curves calculated at the lowest subsample size of 30000 sequences per sample, show the effects of sequencing efforts on the observed number of OTUs at 97% sequence similarity. (b) Good’s coverage indices. C, control group; L, low-dose (0.5 g/kg) group with *K*. *marxianus*; M, medium-dose (1.5 g/kg) group with *K*. *marxianus*; H, high-dose (2.5 g/kg) group with *K*. *marxianus*.
(DOCX)

S1 Table Alpha diversity of ileal microbial community (*n* 8). (DOCX)

## Abbreviations

ABW: average body weight
ADG: average daily gain
ADFI: average daily feed intake
FCR: feed conversion ratio
VH: villus height
VCR: villus height to crypt depth ratio
SEM: standard error of the mean
sIgA: secretory immunoglobulin A
ALP: alkaline phosphatase
SGLT1: sodium glucose cotransporter 1
PepT1: H+-dependent peptide transporter 1
L-FABP: liver fatty acid-binding protein
OTUs: operational taxonomic units
PCA: Principal component analysis
