# Supplementary material for: Effects of Kluyveromyces marxianus supplementation on immune responses, intestinal structure and microbiota in broiler chickens
Source: PLoS One. 2017 Jul 10;12(7):e0180884. doi: 10.1371/journal.pone.0180884 (PMC5507273; doi:10.1371/journal.pone.0180884)
Supplement: S1 Fig — (a) Rarefaction curves calculated at the lowest subsample size of 30000 sequences per sample, show the effects of sequencing efforts on the observed number of OTUs at 97% sequence similarity. (b) Good’s coverage indices. C, control group; L, low-dose (0.5 g/kg) group with K. marxianus; M, medium-dose (1.5 g/kg) group with K. marxianus; H, high-dose (2.5 g/kg) group with K. marxianus. (DOCX) [file pone.0180884.s001.docx]

| a | b |
| --- | --- |
| 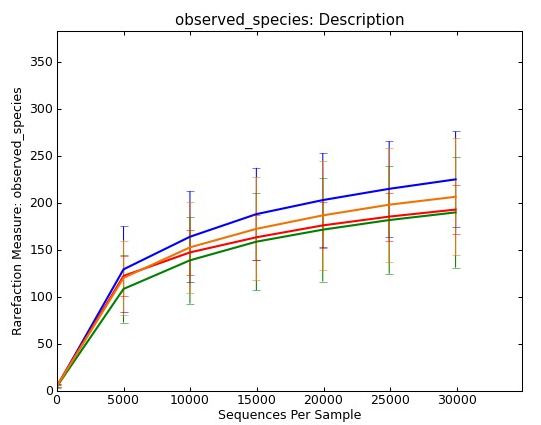  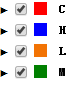 | 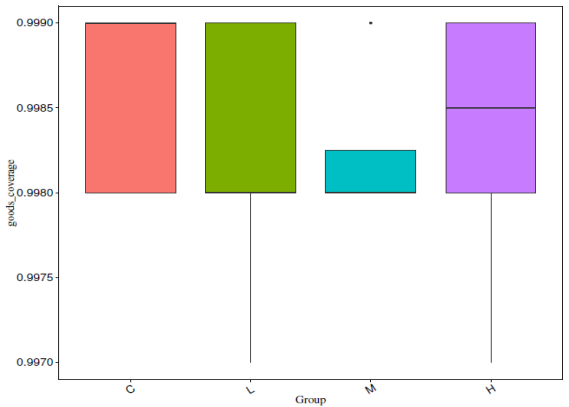 |

**S1 Fig. Rarefaction curves and Good’s coverage of ileal microbial sequencing (*n* 8).** (a) Rarefaction curves calculated at the lowest subsample size of 30000 sequences per sample, show the effects of sequencing efforts on the observed number of OTUs at 97% sequence similarity. (b) Good’s coverage indices. C, control group; L, low-dose (0.5 g/kg) group with *K. marxianus*; M, medium-dose (1.5 g/kg) group with *K. marxianus*; H, high-dose (2.5 g/kg) group with *K. marxianus*.
