# Supplementary material for: Effects of Kluyveromyces marxianus supplementation on immune responses, intestinal structure and microbiota in broiler chickens
Source: PLoS One. 2017 Jul 10;12(7):e0180884. doi: 10.1371/journal.pone.0180884 (PMC5507273; doi:10.1371/journal.pone.0180884)
Supplement: S1 Table — (DOCX) [file pone.0180884.s002.docx]

**S1 Table. Alpha diversity of ileal microbial community**

| Dose  (g/kg) | Shannon index | Simpson index | Chao1 index | ACE index |
| --- | --- | --- | --- | --- |
| 0 (C） | 3.66 | 0.78 | 234.53 | 230.68 |
| 0.5 (L） | 3.48 | 0.80 | 254.24 | 250.69 |
| 1.5 (M) | 3.03 | 0.68 | 239.00 | 238.97 |
| 2.5 (H) | 3.18 | 0.71 | 267.92 | 274.09 |
| SEM | 0.169 | 0.029 | 10.941 | 10.357 |
| *P*-value |  |  |  |  |
| Linear | 0.241 | 0.182 | 0.410 | 0.211 |
| Quadratic | 0.388 | 0.362 | 0.683 | 0.424 |

*n* = 8 replicates per group.
